# Antibacterial Activity of Endophytic Bacteria Against Sugar Beet Root Rot Agent by Volatile Organic Compound Production and Induction of Systemic Resistance

**DOI:** 10.3389/fmicb.2022.921762

**Published:** 2022-06-02

**Authors:** Somayeh Safara, Behrouz Harighi, Bahman Bahramnejad, Slahadin Ahmadi

**Affiliations:** ^1^Department of Plant Protection, Faculty of Agriculture, University of Kurdistan, Sanandaj, Iran; ^2^Department of Plant Production and Genetics, Faculty of Agriculture, University of Kurdistan, Sanandaj, Iran; ^3^Department of Medical Physiology and Pharmacology, School of Medicine, Kurdistan University of Medical Sciences, Sanandaj, Iran

**Keywords:** *Bacillus pumilus*, endophytic bacteria, induce resistance, sugar beet root rot, virulence traits, volatile compounds

## Abstract

The volatile organic compounds (VOCs) produced by endophytic bacteria have a significant role in the control of phytopathogens. In this research, the VOCs produced by the endophytic bacteria *Streptomyces* sp. B86, *Pantoea* sp. Dez632, *Pseudomonas* sp. Bt851, and *Stenotrophomonas* sp. Sh622 isolated from healthy sugar beet (*Beta vulgaris*) and sea beet (*Beta maritima*) were evaluated for their effects on the virulence traits of *Bacillus pumilus* Isf19, the causal agent of harvested sugar beet root rot disease. The gas chromatographymass spectrometry (GC-MS) analysis revealed that B86, Dez632, Bt851, and Sh622 produced 15, 28, 30, and 20 VOCs, respectively, with high quality. All antagonistic endophytic bacteria produced VOCs that significantly reduced soft root symptoms and inhibited the growth of *B. pumilus* Isf19 at different levels. The VOCs produced by endophytic bacteria significantly reduced swarming, swimming, and twitching motility by *B. pumilus* Isf19, which are important to pathogenicity. Our results revealed that VOCs produced by Sh622 and Bt851 significantly reduced attachment of *B. pumilus* Isf19 cells to sugar beetroots, and also all endophytic bacteria tested significantly reduced chemotaxis motility of the pathogen toward root extract. The VOCs produced by Dez632 and Bt851 significantly upregulated the expression levels of defense genes related to soft rot resistance. Induction of *PR1* and *NBS-LRR2* genes in sugar beetroot slices suggests the involvement of SA and JA pathways, respectively, in the induction of resistance against pathogen attack. Based on our results, the antibacterial VOCs produced by endophytic bacteria investigated in this study can reduce soft rot incidence.

## Introduction

Sugar beet (*Beta vulgaris* L.) is one of the most crops for sugar production all over the world (Trebbi and McGrath, 2004). The amount of sugar beet production exceeds 275 million tons per year worldwide and Iran with a production of approximately 5 × 10^6^ tons per year is in the top twenty sugar beet producing countries (Food and Agriculture Organization [FAO], 2020). The sugar beet can be infected by various bacteria both in the field and in storage. Bacterial root rot disease causes serious losses in sugar beet yield. Usually, sugar beet is subjected to storage providing ideal conditions for microbial growth. Therefore, healthy harvested sugar beet can display rot symptoms during the storage period (Liebe and Varrelmann, 2016). Our storage observations and isolations from the most important sugar beet production areas in Iran revealed that several bacteria might play important roles in causing bacterial root rot. Symptoms include dark-brown to black lesions on taproot. Lesions may be superficially restricted to dark areas on the root. In the advanced stage, the nearly entire root may rot and produce viscous slime. Based on partial nucleotide sequencing of the *16S rRNA* gene, one isolate with high sugar beet root rot activity was identified as *Bacillus pumilus* Isf19.

*B. pumilus* is a gram-positive, rod-shaped bacterial species found in healthy plant tissues that promote plant growth by enhancing nutrient uptake, atmospheric nitrogen fixation, reducing metal toxicity, and producing antimicrobial substances against various plant pathogenic microorganisms (Yuan and Gao, 2015). Some *B. pumilus* strains have been previously reported as plant pathogens, including the causal agent of leaf blight of mango trees in Egypt (Galal et al., 2006), the ginger rhizome rot pathogen in China (Peng et al., 2013), *Bacillus* sp., identified as a possible *B. pumilus* strain, bacteria associated with leaf and twig dieback of Asian pear trees in China (Li et al., 2009), and the causative agent of Persian oak decline in Iran (Ahmadi et al., 2019).

Necrotrophic bacteria use different strategies employing the production of plant cell wall-degrading enzymes and necrosis-inducing proteins to kill plant tissues (Davidsson et al., 2013). Several plant cell wall-degrading enzymes, including xylanase (Nagar et al., 2012; Subramaniyan, 2012), pectate lyase (Basu et al., 2009), and β-1,4-endoglucanase (Lima et al., 2005), have been isolated from *B. pumilus* strains that may be related to the mechanisms of pathogenicity. Whole-genome analysis of a ginger rot pathogen *B. pumilus* GR8 revealed that numerous plant cell wall-degrading enzymes and several proteins involved in the interaction between bacterial pathogen and plant are encoded by the genome of this pathogen (Yuan and Gao, 2015).

Plants are usually associated with different microorganisms; among them, endophytic bacteria that colonize the same ecological niche in plants as plant pathogens might have the potential to suppress the virulence of pathogenic microorganisms (Compant et al., 2005). In sugar beet, the diversity and ecology of the endophytic bacteria have been investigated. Research on endophytic bacteria of sugar beet has included the identification of phyla *Proteobacteria, Actinobacteria*, and *Firmicutes* (Wolfgang et al., 2020). Bacterial isolates, such as *Pseudomonas* sp., *Pantoea agglomerans, Microbacterium testaceum*, and *Subtercola pratensis*, have been identified as demonstrating antagonistic potential against various fungal plant pathogens under *in vitro* conditions (Zachow et al., 2008).

Bacteria have been reported to emit various volatile compounds with significant biological activities on a broad range of plant pathogens (Garbeva and Weisskopf, 2020). In general, volatile compounds are defined as small molecules (<300 Da) of low boiling point with high vapor pressure. These molecules can spread easily between roots and microbes even at distances (Zarraonaindia et al., 2015). The chemical structures of bacterial volatiles are very diverse so that in different groups like small aliphatic, aromatic molecules to large molecules are observed (Schulz and Dickschat, 2007). A few investigations have been reported regarding the antibacterial activity of these volatile compounds (Rajer et al., 2017; Xie et al., 2018). *Pseudomonas fluorescens* B-4117 and *Serratia plymuthica* IC1270 produced VOCs that reduced the growth of *Agrobacterium tumefaciens* and *Agrobacterium vitis* (Dandurishvili et al., 2011). Volatile organic compounds released by *Bacillus amyloliquefaciens* SQR-9 and *Pseudomonas fluorescens* WR-1 can inhibit the growth and virulence traits of *Ralstonia solanacearum* (Raza et al., 2016a,b). VOCs produced by *Bacillus* strains caused morphological abnormalities in *R. solanacearum* cells (Tahir et al., 2017).

Plants generally enhance local or systemic resistance to pathogens or biotic stress. These responses are generally mediated by jasmonic acid (JA) and salicylic acid (SA) pathways which lead to the enhancement of plant defense genes, such as the nucleotide-binding site–leucine-rich repeat (*NBS-LRR2*) or pathogenesis-related (*PR*) genes, respectively (Aswani et al., 2022). *NBS-LRR* gene-mediated disease resistance is among the most important plant defense mechanisms against pathogens (Marone et al., 2013). A previous study revealed that sugar beetroots treated with jasmonic acid have improved resistance to the storage pathogens like *Botrytis cinerea*, *Penicillium claviforme*, and *Phoma betae* (Fugate et al., 2012). Jasmonic acid induces defense responses and upregulated defense genes, including *NBS-LRR* family, in harvested sugar beetroots (Fugate et al., 2017). Unfortunately, to the best of our knowledge, the roles of *NBS-LRR2* gene related to sugar beet soft rot disease resistance have not been investigated. *PR1* gene expression is induced in response to a variety of pathogens. Some PR proteins with chitinase and β-1,3-glucanase activity have been identified in sugar beet as a defense reaction to *Cercospora beticola* (Weltmeier et al., 2011). However, little is known about the impact of *B. pumilus* as a postharvest root rot agent on the expression of *PR* genes. Endophytic bacteria can protect their plant hosts against a wide range of pathogens through triggering defense reactions (Pieterse et al., 2012). Previous studies have shown that some endophytic bacteria activate plant defense reactions against biotrophic and necrotrophic plant pathogens. In some pathosystems, induction of defense reactions was mediated by the upregulation of *PR* genes (Asghari et al., 2020; Portieles et al., 2021).

To the best of our knowledge, there is no research about the effect of VOCs produced by endophytic bacteria on the virulence traits of *B. pumilus*. Therefore, the objectives of this study were to evaluate the effects of VOCs produced by endophytic bacteria as biocontrol agents on the growth rate, and virulence traits, such as motility, chemotaxis, attachment, and biofilm formation of sugar beet soft rot pathogen *B. pumilus*. The major VOCs produced by endophytic bacteria against *B. pumilus* were identified using gas chromatography–mass spectrophotometry (GC-MS). Also, the activity of selected endophytic bacteria to induce defense responses in sugar beet roots against the bacterial pathogen was investigated.

## Materials and Methods

### Bacterial Strains and Growth Conditions

*Bacillus pumilus* Isf19 (GenBank Acc. No MZ647525) which exhibited soft rot disease in harvested sugar beetroot, as well as the endophytic bacteria *Streptomyces* sp. B86 (GenBank Acc. No. MZ647528), *Pantoea* sp. Dez632 (GenBank Acc. No. MZ647535), *Pseudomonas* sp. Bt851 (GenBank Acc. No. MZ647537), and *Stenotrophomonas* sp. Sh622 (GenBank Acc. No. MZ647534) isolated from sugar beet and sea beet plants provided by our laboratory were used in this study. These strains were routinely grown on nutrient agar (NA) medium and stored at 4–6°C as a working stock. All strains were grown in nutrient broth (NB) medium for 24 h at 26–28°C with shaking, and sterile glycerol was added to the final concentration of 20% and then stored at −20°C for long-term storage.

### Effect of Volatile Organic Compounds on Soft Rot Development by Bacterial Pathogen

The healthy sugar beetroots were immersed in 0.5% sodium hypochlorite solution for 3 min, rinsed in sterile-distilled water, and allowed to air-dry under sterile conditions. All exterior parts of the root were removed, and then root slices of about 0.5-cm thick and 3–4 cm in diameter were placed in one compartment of divided plates. A hole (5-mm depth) was made in the center of the slice, and 40 μl of the freshly prepared bacterial pathogen suspension (density of about 1 × 10^8^ CFU ml^–1^) was added. In the other compartment, 40 μl of each endophytic bacteria with a concentration of about 1 × 10^11^ CFU ml^–1^ was streaked on the NA medium. The plates were sealed with parafilm and incubated at 30–32°C, and the diameter of soft rotted area was measured for up to 7 days (Strausbaugh and Gillen, 2008). Root slices inoculated with sterile water and only pathogen were used as negative and positive controls, respectively.

### Antibacterial Activity of Volatile Organic Compounds Produced by Endophytic Bacteria

The antibacterial activity of VOCs produced by endophytic bacteria against bacterial pathogen was assessed on NA medium using a dual-culture technique. The 24 h growth of the endophytic bacteria (adjusted to the concentration of about 1 × 10^11^ CFU ml^–1^) was cultured on one side of the plate, while the opposite side was spot inoculated with 5 μl of the pathogen (1 × 10^8^CFU ml^–1^). In the control, the pathogen was cultured alone. The plates were then sealed with parafilm and maintained at 28°C for 7 days. The colony numbers of the *B. pumilus* Isf19 were calculated (Tahir et al., 2017). Three replications were performed for each treatment.

### Effect of Volatile Organic Compounds on Swarming, Swimming, and Twitching Motility Behaviors of Bacterial Pathogen

The various motility behaviors of the bacterial pathogen cells exposed to VOCs of endophytic bacteria were assessed using a divided Petri plate. Two microliters of the freshly prepared bacterial pathogen (which was adjusted to the concentration of about 1 × 10^8^CFU ml^–1^) was spotted on one compartment of the divided plates containing LB medium plus agar (0.3, 0.7, and 1.6%) for swimming, swarming, and twitching motility, respectively. In the other compartment, 20 μl of the endophytic bacterial suspension (concentration of about 1 × 10^11^ CFU ml^–1^) was cultured on the NA medium. The plates were incubated at 26–28°C, and the colony diameter of swarming and swimming motility was measured each 12-h interval. The halo diameter of twitching motility was examined after 72 h. Notably, the experiments were done in three replications (Tahir et al., 2017).

### Chemotaxis Assay

The overnight growth of the endophytic bacteria was streaked onto one compartment of divided plates containing NA medium. In the other compartment, chemotaxis buffer medium (0.1 mM EDTA, 10 mM K_2_HPO_4_, 0.05% glycerol, 5 mM lactic acid, 0.14 μM CaCl_2_, 0.3 mM (NH_4_)_2_SO_4_, 0.35% agar, and pH 7.2) was prepared (Ordal and Gibson, 1977). Thereafter, 10 mm of the medium was removed and then refilled with 100 μl of root extract of sugar beet (var. Ekbatan). Then, 5 μl of bacterial pathogen cells was spot inoculated at a distance of 5 mm from the hole. The plates were sealed with parafilm and incubated at 28°C. The movement of the bacterial pathogen cells toward the root extract was counted as the CFU ml^–1^ of the cell. For the preparation of root extract, 2 g of sugar beet roots was homogenized into 20 ml of sterile 0.1 M phosphate buffer, transferred to a sterile 50-ml falcon tube, and incubated for 24 h. Samples were then filtered using a 0.22 μm Millipore membrane filter and stored at −20°C. This experiment was performed in three replicates.

### Biofilm Formation Assay

The biofilm formation property of bacterial pathogen cells exposed to VOCs was investigated in polypropylene tubes. Accordingly, twenty microliters of the freshly prepared culture of endophytic bacterial strains (about 1 × 10^11^CFU ml^–1^) was cultured onto one compartment of the divided plates containing NA medium. In the other compartment, a microtube containing 150 μl of LB liquid medium plus 1% glucose that was inoculated with 50 μl of the bacterial pathogen (1 × 10^8^ CFU ml^–1^) was placed vertically. The plates were then sealed with parafilm and maintained at 26–28°C. After 48–72 h, bacterial cells were discarded and 150 μl of 1% crystal violet solution was added to each tube and kept at room temperature for 15 min. The microtubes were then washed twice with sterile water. Subsequently, 2 × 200 μl of 96% ethanol was added to each microtube, the resulting volume was brought to 1 ml with sterile-distilled water, and the absorbance was measured at 540 nm with a spectrophotometer (SPECORD 210, Analytik Jena, Germany). Non-exposed bacterial pathogen cells to VOCs were used as a control. The experiment was conducted in a completely randomized design with three replications (O’Toole and Kolter, 1998).

### Sugar Beet Root Attachment Assay

The attachment of bacterial pathogen cells to the root of sugar beet plantlets was assayed after exposure to VOCs produced by endophytic bacteria for 72 h. The roots of forty-day-old sugar beet plantlets (var. Ekbatan) were submerged in 10 ml treated or untreated bacterial pathogen cell suspensions (adjusted to about 1 × 10^8^CFU ml^–1^) at room temperature and after 3 h rinsed three times with sterile-distilled water. Then, 3–5 mm from the root tips were separated, weighed, and placed individually in 1 ml of sterile-distilled water. After stirring for 5 s, the roots were macerated in 100 μl of sterile water. The obtained suspension was streaked onto NA medium and incubated at 28°C for 48 h, and the CFU ml^–1^ was counted. The experiment was conducted with three replications.

### Effect of Volatile Organic Compounds on Cell Morphology

Scanning electron microscopy (SEM) was used to observe external morphological abnormalities of the bacterial pathogen cells. Bacterial cells with or without exposure to the VOCs of endophytic bacteria for 7 days at 30–32°C were collected into Eppendorf tubes and washed twice with 0.1 M phosphate buffer saline (PBS, pH:7.2). Afterward, the cells were spread onto the aluminum foil placed on the clean slide. After fixation in 2.5% glutaraldehyde solution for 1 h at room temperature, the samples were washed three times with PBS. Serial dehydration was done in ethanol solutions of 35, 50, 75, 90, and 100%, for 5 min, each time followed by 100% ethanol for 1 h. The samples were then conducted by the freeze-drying process at −70°C for 3 h. Finally, the samples were coated with gold, and electron micrographs were taken using a Philips XL30 SEM system (Philips SEM, Netherland).

### Gene Expression Analysis of Inoculated Roots

The expression of the *PR1* and *NBS-LRR2* genes as a marker of salicylic acid (SA) and jasmonic acid (JA) pathways, respectively, was measured by qRT-PCR in the root slices of sugar beet. Treated roots with Dez632 and Bt851 that showed a higher reduction in soft rot development, also roots inoculated with Dez632/Isf19, Bt851/Isf19, and untreated roots (control) were collected after 0, 2, and 7 days, placed in aluminum foil, and stored in a sterile microtube at −40°C until used. For extraction of total RNA, 0.5 g of frozen powdered tissues was macerated into 1 ml of extraction buffer (per TE buffer pH:8.0 of 13 ml saturated phenol, 0.32 M sodium acetate, 0.01 M EDTA, 1% SDS, 1% PVP, and 3% CTAB). The suspension was centrifuged (8,000 rpm, 5 min, 4°C), and the resulting supernatant was transferred into a new tube. The supernatant was mixed with an equal volume of phenol/chloroform/isoamyl alcohol (25/24/1) and centrifuged, and RNA was precipitated with 0.1 volume 3M sodium acetate (pH:5.0) and an equal volume of isopropanol overnight at −20°C. Finally, the suspension was centrifuged at 13,500 rpm for 15 min, and RNA was washed with 70% ethanol, dried, and dissolved in 50 μl of RNase-free-DEPC water. The concentration and purity of RNA were determined using NanoDrop (Thermo Fisher Scientific, United States). DNase treatment was performed using the RNase-free DNase I (Yekta Tajhiz Azma, Iran).

cDNA was amplified from 200 ng of total RNA using the cDNA synthesis Kit (Parstous, Iran) according to the manufacturer’s instructions. Real-time PCR (RT-PCR) was performed with RealQ Plus 2x Master Mix Green (Ampliqon, Denmark) using an AB Applied Biosystem StepOne thermal cycler. PCR reactions were carried out in a 10 μl final volume containing 2x Master Mix Green High ROX, 5 p.m. forward and reverse primers (Table 1), and cDNA. Cycling conditions were 10 min at 95°C, followed by 40 two-step cycles of 15 s at 95°C and 1 min at 60°C. β*-actin* gene was used as a reference gene for normalization. The specificity of each primer was checked using the method previously described (Schmittgen and Livak, 2008). Relative gene expression was calculated with the following formula (Pfaffl, 2001):


$$\mathrm{Relative}\,\mathrm{expersion}=e^{-\Delta \,\mathrm{Ct}}=e^{-\left(\mathrm{Ct}\,\mathrm{target}\,\mathrm{gene}-\mathrm{Ct}\,\mathrm{refrence}\,\mathrm{gene}\right)}$$

**TABLE 1:** Primer sequences used for RT-PCR analysis of defense-related genes.

	Primer sequences	References
*PR-1*	5′-CAAGTAGTGTGGAGAGAATCGG-3′ 5′-TGTAATTGCCAGGAGGATCATAA-3′	Schmidt et al., 2020
*NBS-LRR2*	5′-GGGTAAGAGAGTTGCCAAGC-3′ 5′-TCCACAAGTGCAGAAGTTCG-3′	Fugate et al., 2017
*B -actin*	5′-GATTTGGCACCACACCTTCT-3′ 5′-TCTTTTCCCTGTTTGCCTTG-3′	Fugate et al., 2017

The Ct values were the means of three biological replications and three technical replications.

### Identification of Volatile Organic Compounds Produced by Endophytic Bacteria Using Gas Chromatographymass Spectrometry Analysis

To collect the VOCs produced by each endophytic bacteria, a three-compartment plate was used. One compartment, containing NA medium, was streaked with 20 μl (1 × 10^11^ CFU ml^–1^) overnight growth of each endophytic bacteria, a second compartment, containing NA medium, was spot inoculated with 5 μl (1 × 10^8^ CFU ml^–1^) of *B. pumilus* Isf19, and the third compartment was filled with 0.3 g of sterile activated charcoal to adsorb the VOCs. The same experimental design, including endophytic bacteria and bacterial pathogen without activated charcoal, and also bacterial pathogen alone was used as control. The plates were sealed with parafilm and incubated at 28–30°C for 72 h. The activated charcoal traps were transferred into glass vials, and ethyl acetate (1: 1.25 W/V) was added. The adsorbed VOCs were extracted by shaking for 15 min, followed by centrifugation (5,000 rpm, 15 min), and the supernatants were analyzed by a gas chromatography device connected to a mass spectrometer (Agilent 7890B GC System/5977A MSD, Agilent Technologies, United States).

One microliter of the sample was injected into HP-5 ms column, and the initial column temperature was 50°C, which was increased to 240°C at a rate of 5°C min^–1^, held for 5 min. The mass spectrometer was operated in the electron ionization mode at 70 eV, with continuous scanning from 50 to 550 m/z. Helium carrier gas with a purity of 99.999%, a 34 psi pressure, and a flow rate of 1 ml min^–1^ was used. The compounds were identified by comparing their mass spectra with the databases of the device, including the National Institutes of Standards and Technology (NIST) and Wiley databases.

### Statistical Analysis

All experiments were conducted in a completely randomized design. To evaluate the significance of the treatments, the data from each experiment were analyzed using analysis of variance (ANOVA), followed by the least-significant difference (LSD) test (*P* = 0.05), employing SAS ver. 9.1 statistical software (SAS, 2009). Graphs were plotted using the Excel program.

## Results

### Effect of Volatile Organic Compounds on Soft Rot Disease Development and Their Antibacterial Activity

**FIGURE 1:**
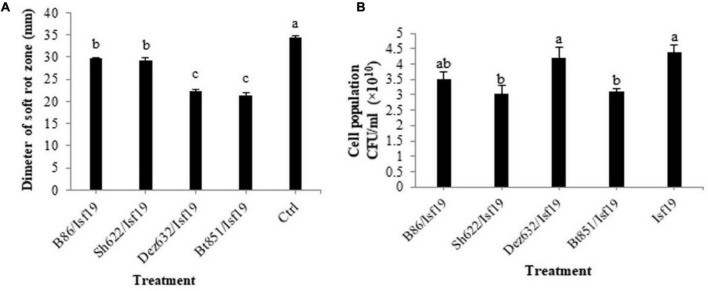
Effects of VOCs produced by *Streptomyces* sp. B86, *Pantoea* sp. Dez632, *Pseudomonas* sp. Bt851, and *Stenotrophomonas* sp. Sh622 on **(A)** soft rot development by *B. pumilus* Isf19 in sugar beetroot slices compared to the non-treated control (Ctrl), and **(B)** cell population of *B. pumilus* Isf19. Three replicates were used for each treatment. Error bars indicate the SE of the three replicates. Different letters indicate significant differences (*P* = 0.05).

The results showed that significant differences existed between treatments in the reduction in soft rot development (*F* = 79.03; *P* < 0.0001) compared to the control (Table 2). All endophytic bacteria significantly reduced the soft rot symptoms produced by *B. pumilus* Isf19 at varying levels. VOCs produced by strains Bt851 and Dez632 decreased the symptom development to about 38 and 35%, respectively, followed by Sh622 and B86 with a 15% reduction effect (Figure 1A).

**TABLE 2:** Analysis of variance (ANOVA) of swimming, swarming, twitching motility, biofilm production, cell population, chemotaxis, attachment, and soft rot development by *B. pumilus* Isf19 under the effect of VOCs produced by endophytic bacteria.

		Mean of square							
Source of variation	df	Swimming	Swarming	Twitching	Biofilm	Cell population	Chemotaxis	Attachment	Soft rot development
Treatment	4	161.85**	398.62**	0.90**	0.03**	0.67**	663485.06**	141407.70**	89.56**
Error	10	0.45	12.35	0.05	0.008	0.17	29678.13**	1926.66	1.13
*F*-value		359.67	32.28	18.00	3.85	22.36	359.67	73.40	79.03

**Significant at 5% probability level.

According to statistical analysis, significant differences existed between treatments in the reduction in the cell population of *Bacillus* sp. Isf19 (*F* = 3.85; *P* = 0.0304) exposed to VOCs produced by endophytic bacteria compared to the non-exposed control (Table 2). VOCs of Sh622 and Bt851 strains lead to significant cell population inhibition of *B. pumilus* Isf19 to about 30%, followed by B86 with a 21% reduction effect (Figure 1B).

### Effect of Volatile Organic Compounds on the Motility Behaviors of *B. pumilus* Isf19

**FIGURE 2:**
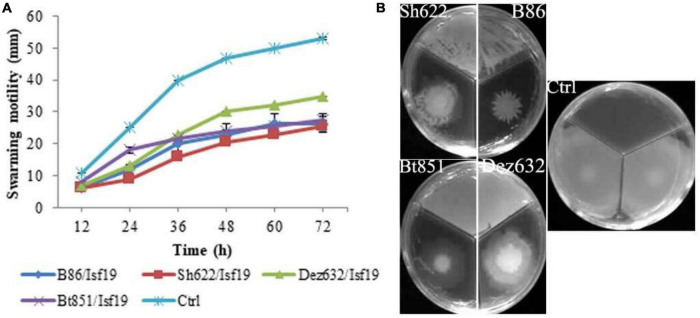
Effects of VOCs produced by *Streptomyces* sp. B86, *Pantoea* sp. Dez632, *Pseudomonas* sp. Bt851, and *Stenotrophomonas* sp. Sh622 on swarming motility of *B. pumilus* Isf19 compared to the non-treated control (Ctrl). The diameter of motility zone **(A)** and representative plate of swarming motility assay **(B)** were shown. Three replicates were used for each treatment. Error bars indicate the SE of the three replicates. Different letters indicate significant differences (*P* = 0.05).

According to statistical analysis, significant differences existed between treatments in the swarming (*F* = 32.28; *P* < 0.0001), swimming (*F* = 359.67; *P* < 0.0001), and twitching (*F* = 18.00; *P* = 0.0001) assays (Table 2). Our finding revealed that the swarming motility of *B. pumilus* Isf19 was significantly inhibited during 72 h exposure to VOCs of endophytic bacterial strains. As illustrated in Figures 2A,B, strains Sh622, B86, and Bt851, with a mean of 25.5, 26.5, and 27.5 mm, respectively, showed the highest inhibition effects of *B. pumilus* Isf19 cells followed by Dez632 with a mean of 35 mm, as compared to the control with the mean of 53 mm.

**FIGURE 3:**
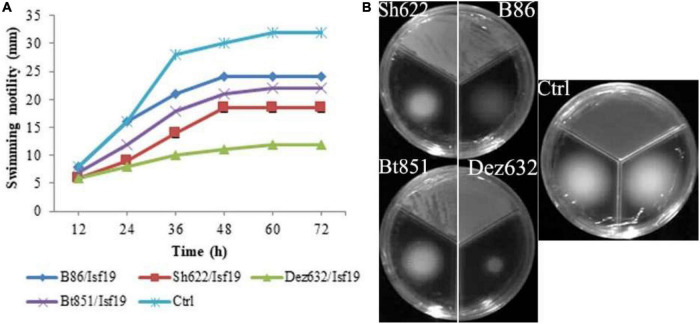
Effects of VOCs produced by *Streptomyces* sp. B86, *Pantoea* sp. Dez632, *Pseudomonas* sp. Bt851, and *Stenotrophomonas* sp. Sh622 on swimming motility of *B. pumilus* Isf19 compared to the non-treated control (Ctrl). The diameter of motility zone **(A)** and representative plate of swarming motility assay **(B)** were shown. Three replicates were used for each treatment. Error bars indicate the SE of the three replicates. Different letters indicate significant differences (*P* = 0.05).

**FIGURE 4:**
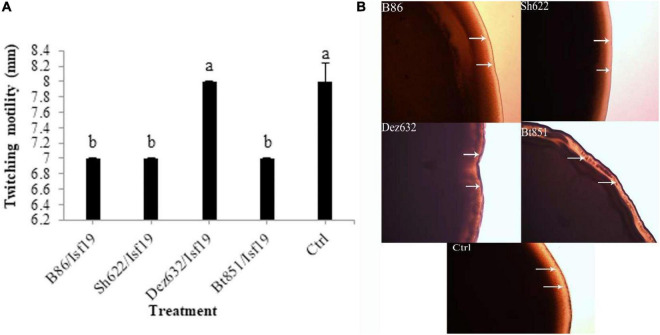
Effects of VOCs produced by *Streptomyces* sp. B86, *Pantoea* sp. Dez632, *Pseudomonas* sp. Bt851, and *Stenotrophomonas* sp. Sh622 on twitching motility of *B. pumilus* Isf19 compared to the non-treated control (Ctrl). The diameter of motility zone **(A)** and representative plate of swarming motility assay **(B)** were shown. Three replicates were used for each treatment. Error bars indicate the SE of the three replicates. Different letters indicate significant differences (*P* = 0.05).

The VOCs produced by Dez632 and Sh622 inhibited swimming motility of *B. pumilus* Isf19 to 12 and 18.5 mm, respectively, as compared to control with 32 mm and had the highest negative effect after 72 h (Figures 3A,B). Similarly, twitching motility was significantly reduced to 7 mm by B86, Sh622, and Bt851 compared to control with 8 mm (Figure 4A). Microscopic examination of the twitching motility exhibited that the circumferential colony edge of *B. pumilus* Isf19 in the non-exposed control was significantly wider than those exposed with the Dez632 and Bt851 volatiles (Figure 4B).

### Effect of Volatile Organic Compounds of Endophytic Bacteria on Chemotaxis, Biofilm Formation, and Root Attachment

**FIGURE 5:**
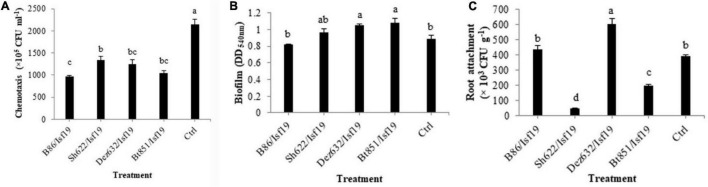
Effects of VOCs produced by *Streptomyces* sp. B86, *Pantoea* sp. Dez632, *Pseudomonas* sp. Bt851, and *Stenotrophomonas* sp. Sh622 on **(A)** chemotaxis behavior toward root extract of sugar beet, **(B)** biofilm formation, and **(C)** root attachment of *B. pumilus* Isf19 compared to the non-treated control (Ctrl). Error bars indicate the SE of the three replicates. Different letters indicate significant differences (*P* = 0.05).

Based on the results of ANOVA analysis, significant differences existed between all treatments in the chemotaxis assay in the number of cells migrated toward the sugar beetroot extract (*F* = 22.36; *P* < 0.0001) (Table 2). As shown in Figure 5A, VOCs produced by B86, Bt851, Dez632, and Sh622 strains by about 55, 52, 43, and 38%, respectively, reduced the motility of *B. pumilus* Isf19 cells toward root extract compared with control.

As presented in Table 2, the result of ANOVA showed a significant difference between all treatments in the biofilm formation assay compared with non-treated control (*F* = 4.31; *P* = 0.0277). As shown in Figure 5B, no significant difference was observed in the biofilm formation by *B. pumilus* Isf19 exposed to VOCs of B86 and Sh622. Interestingly, significant increasing effects in biofilm formation were observed after treatment of *B. pumilus* Isf19 with volatiles of Dez632 and Bt851 strains compared to the control.

The ability of *B. pumilus* Isf19 to attach to sugar beetroot segments after treatment with endophytic bacterial strains was investigated. Based on the results of ANOVA, significant differences existed between all treatments in the root attachment of treated *B. pumilus* Isf19 cells (*F* = 73.40; *P* < 0.0001). The highest inhibition was related to Sh622 with 88% reduction and then was observed for Bt851 with 50% reduction. Furthermore, our findings showed no significant reduction in the attachment of exposed *B. pumilus* Isf19 cells to roots by B86 compared with control (Figure 5C). Surprisingly, volatiles of Dez632 increased the root attachment of *B. pumilus* Isf19 cells.

### Scanning Electron Microscopy

**FIGURE 6:**
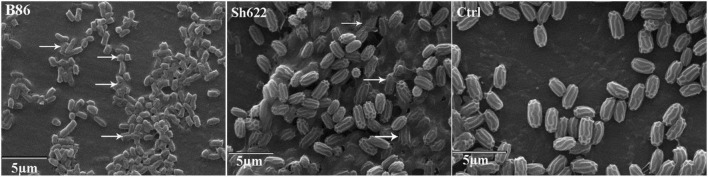
Scanning electron micrographs of *Bacillus pumilus* Isf19 cells exposed with VOCs produced by *Streptomyces* sp. B86 and *Stenotrophomonas* sp. Sh622 strains. Ctrl: non-treated control cells. Arrowheads indicate cell disruption or abnormality.

SEM analysis revealed that more than 70 and 30% of the *B. pumilus* Isf19 cell exposure to the VOCs produced by *Streptomyces* sp. B86 and *Stenotrophomonas* sp. Sh622 strains, respectively, showed a wide range of morphological abnormalities compared to the non-exposed control (Figure 6). The SEM of the non-treated control showed normal cell shape and growth. By contrast, the *B. pumilus* Isf19 cells were damaged in the presence of the VOCs.

### Induction of Defense Responses in Sugar Beetroots

**FIGURE 7:**
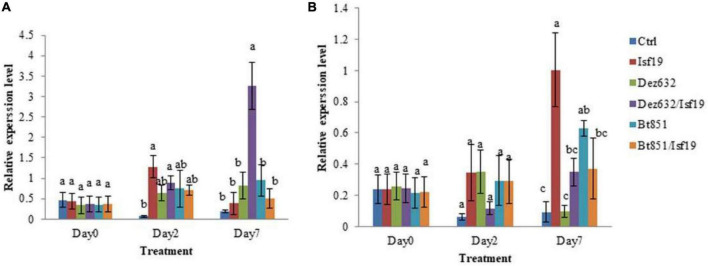
Relative expression levels of *PR1* **(A)** and *NBS-LRR2* **(B)** defense-related genes in the non-inoculated sugar beet root slices (Ctrl), root slices treated by *Pantoea* sp. Dez632, *Pseudomonas* sp. Bt851, and/or *B. pumilus* Isf19. Results represent the means of three replicates. Vertical bars indicate standard errors (SE), and different letters at each day after treatment indicate statistically significant differences between treatments at probability levels of 5%.

qRT-PCR was conducted to examine the relative expression levels of *PR1* and *NBS-LRR2* genes in the non-inoculated sugar beetroot slices (control) and root treated by Dez632, Bt851, and/or *B. pumilus* Isf19. Results revealed that the expression of the defense gene responsive to salicylic acid (SA), including *PR1*, did not change in sugar beetroot slices treated with endophytic bacteria and pathogen at day 0, but significant differences were observed between all treatments and control two days after inoculation. Furthermore, on Day 7 after inoculation, significant difference was observed regarding *PR1* gene expression level in root slices treated by Dez632 after pathogen challenge (Figure 7A). The expression level of the *PR1* gene increased by 8.5- and 18-fold in Dez632/Isf19 treatment than root slices inoculated with pathogen alone and control, respectively.

In addition, the expression level of the defense gene responsive to jasmonic acid (JA) pathway, including the *NBS-LRR2* gene remained unchanged in sugar beetroot slices treated with endophytic bacteria and/or pathogen at Day 0; however, significant differences were observed between all treatments except Dez632/Isf19 compared to the control 2 days after inoculation. Results revealed that on Day 7 after inoculation, significant difference was observed regarding *NBS-LRR2* gene expression level in root slices treated by endophytic bacteria and/or pathogen compared to the control (Figure 7B). The exception was treated root with Dez632 alone which remains unchanged. The expression of *NBS-LRR2* gene in the root slices inoculated with pathogen alone showed the highest level 7 days after inoculation.

**TABLE 3:** Volatile organic compounds produced by B86, Sh622, Dez632, and Bt851 strains against *Bacillus pumilus* Isf19 and detected by GC–MS analysis.

Volatile organic compounds	*Streptomyces* sp. B86		*Stenotrophomonas* sp. Sh622		*Pantoea* sp. Dez632		*Pseudomonas* sp. Bt851	
	RT (min)	RPA (%)	RT (min)	RPA (%)	RT (min)	RPA (%)	RT (min)	RPA (%)
Ethylbenzene	−	−	−	−	4.29	2.18	4.28	2.00
1-Butanol, 3− methyl-, acetate	4.21	0.75	4.20	0.77	−	−	−	−
2,4-Octadiene	−	−	−	−	−	−	4.43	6.63
p-Xylene	4.58	0.31	4.58	0.90	4.44	10.61	−	−
1-Cycloocten-5-yne, (Z) -	−	−	−	−	−	−	5.06	2.93
Benzene, (1-methylethyl) -	−	−	−	−	5.61	1.03	5.60	0.67
Decane	−	−	4.99	1.39	5.69	2.07	5.69	1.87
Hexanoic acid, 5- oxo-, ethyl ester	−	−	−	−	6.06	0.32	−	−
Benzene, propyl-	−	−	−	−	6.36	3.45	6.36	1.26
Benzene, 1-ethyl-3-methyl-	5.82	0.48	5.73	1.74	6.62	10.56	−	−
Benzene, 1,2,3-trimethyl-	−	−	−	−	−	−	6.80	3.50
2,3-Heptadien-5-yne, 2,4-dimethyl	−	−	−	−	6.80	5.87	7.56	1.48
Decane, 2,4-dimethyl-	−	−	−	−	6.91	1.18	−	−
Benzene, 1-ethyl-2-methyl-	−	−	−	−	7.22	1.44	6.62	8.41
Benzene, 1,2,4-trimethyl-	−	−	−	−	7.57	2.33	−	−
Octane, 3,4,5,6-tetramethyl-	−	−	−	−	−	−	8.09	0.88
Acetophenone	−	−	−	−	8.55	0.37	−	−
2-Tolyloxirane	−	−	−	−	8.99	0.51	−	−
Benzene, 1-methyl-4-(1-methylethyl) -	−	−	−	−	9.27	0.69	7.22	1.33
Hexane, 1-propoxy-	−	−	−	−	9.72	0.26	−	−
Oxalic acid, ethyl isohexyl ester	−	−	−	−	9.95	0.50	−	−
2-hexyl-1-decanol	10.23	45.90	−	−	−	−	−	−
6-Tetradecanesulfonic acid, butyl ester	−	−	10.50	4.60	−	−	−	−
Decane, 2,9-dimethyl-	−	−	−	−	10.64	0.56	−	−
Dodecane	8.59	0.86	8.57	2.79	11.07	5.03	11.06	4.21
Dodecane, 2-methyl-	−	−	−	−	11.45	0.41	−	−
2-Aminoethylethyl sulfide	−	−	−	−	−	−	11.57	1.73
Silane, cyclohexyldimethoxymethyl-	−	−	−	−	11.57	3.10	−	−
Benzaldehyde, 2,5-dimethyl	−	−	11.85	1.27	−	−	−	−
2,6-Dimethylbenzaldehyde	11.84	0.82	11.95	0.53	−	−	−	−
Sulfurous acid, hexyl heptyl ester	−	−	−	−	11.85	0.71	−	−
Decane, 2,4,6-trimethyl-	−	−	−	−	12.03	0.72	−	−
Dodecane, 4,6-dimethyl-	−	−	−	−	12.19	0.40	−	−
Hexadecane	13.13	2.55	−	−	12.46	1.62	−	−
3-Ethyl-3-methyldecane	−	−	−	−	−	−	12.46	1.66
Undecane, 2-methyl-	12.62	31.04	−	−	−	−	12.69	5.03
Pentadecane	−	−	−	−	12.70	4.61	−	−
Dodecane, 2,7,10-trimethyl-	−	−	9.60	1.52	12.92	0.71	18.21	9.53
n-Hexyl ether	−	−	−	−	−	−	12.91	0.64
Decane, 3,3,8-trimethyl-	−	−	−	−	−	−	13.03	0.51
Dodecane, 2,6,10-trimethyl−	−	−	10.23	23.57	13.05	0.62	−	−
Tetradecane, 2,6,10-trimethyl-	−	−	13.06	1.24	−	−	−	−
S-Methyl methanethiosulphonate	−	−	−	−	13.25	6.52	−	−
Methyl 3-hydroxytetradecanoate	13.70	0.55	−	−	−	−	−	−
Decane, 2,3,5-trimethyl-	−	−	−	−	−	−	13.83	0.95
Octadecane, 2-methyl-	−	−	13.98	1.08	−	−	−	−
Octadecanoic acid, 3- hydroxy-, methyl ester	13.95	0.39	13.72	1.36	−	−	−	−
Benzene, 1,3-bis (1,1-dimethylethyl)-	−	−	−	−	−	−	14.12	8.47
Tetrahydrofuran, 2-ethyl-5-methyl-	−	−	−	−	−	−	14.34	0.52
Hexane, 1-propoxy-	−	−	−	−	−	−	14.57	0.45
Tridecane	−	−	−	−	−	−	16.34	0.96
Heptadecane, 2-methyl-	16.48	1.07	13.15	4.97	−	−	−	−
Tetradecane	12.05	1.08	12.06	3.90	−	−	16.51	3.22
Nonadecane	15.33	1.02	15.39	1.59	−	−	−	−
Undecane, 4,7-dimethyl-	−	−	−	−	−	−	17.67	0.90
Hexyl octyl ether	−	−	−	−	−	−	19.25	0.69
Decane, 2,6,6-trimethyl-	−	−	−	−	−	−	19.49	2.71
Heptane, 2,2,3,3,5,6,6-heptamethyl-	−	−	−	−	−	−	21.56	0.97
Phenol, 3,5-bis (1,1-dimethylethyl)-	−	−	16.05	1.90	−	−	−	−
Phenol, 2,4-bis (1,1-dimethylethyl)-	15.90	3.04	16.10	3.13	−	−	22.17	14.28
Dodecane, 2,6,11-trimethyl-	9.60	0.57	12.64	33.13	−	−	23.35	7.25
Hexacosane	−	−	16.61	2.60	−	−	−	−

*RT, retention time; RPA, relative peak area; -, no VOCs detected.*

### Identification of Volatile Organic Compounds Produced by Endophytic Bacteria

The GC–MS analysis showed that all endophytic bacteria produced VOCs with various profiles (Table 3). Strains B86, Sh622, Dez632, and Bt851 produced 15, 20, 28, and 30 VOCs, respectively, with high quality. The VOCs dodecane was produced by all bacterial strains tested. The VOCs 1-decanol, 2- hexyl-, methyl 3-hydroxytetradecanoate are specifically produced by strain B86. The main VOC produced by this strain was 1-decanol, 2- hexyl-, and undecane, 2-methyl- with peak areas of 45.90% (RT = 10.23) and 31.04% (RT = 12.62), respectively, in the high quality.

Strain Sh622 specifically produced 6-tetradecanesulfonic acid, butyl ester, benzaldehyde, 2,5-dimethyl, tetradecane, 2,6,10- trimethyl-, octadecane, 2- methyl-, phenol, 3,5-bis (1,1-dimethylethyl)-, and hexacosane volatiles. The main VOCs produced by Sh622 were dodecane, 2,6,10-trimethyl- with a peak area of 23.57% (RT = 10.23) and dodecane, 2,6,11-trimethyl- with a peak area of 33.13% (RT = 12.64).

Only strain Dez632 produced hexanoic acid, 5- oxo-, ethyl ester, decane, 2,4- dimethyl-, benzene, 1,2,4- trimethyl-, acetophenone, 2-tolyloxirane, hexane, 1- propoxy-, oxalic acid, ethyl isohexyl ester, decane, 2,9- dimethyl-, dodecane, 2- methyl-, silane, cyclohexyldimethoxymethyl-, sulfurous acid, hexyl heptyl ester, decane, 2,4,6- trimethyl-, dodecane, 4,6- dimethyl-, pentadecane, and S-methyl methanethiosulfonate as VOCs. The main VOCs produced by this strain were p-xylene and benzene, 1-ethyl-3-methyl- with peak areas of 10.61% (RT = 4.44) and 10.56 (RT = 6.62), respectively.

Strain Bt851 specifically produced VOCs 2,4-octadiyne, 1-cycloocten-5-yne, (Z)-, benzene, 1,2,3- trimethyl-, octane, 3,4,5,6- tetramethyl-, 2-aminoethylethyl sulfide, 3-ethyl-3-methyldecane, n-hexyl ether, decane, 3,3,8- trimethyl-, decane, 2,3,5- trimethyl-, benzene, 1,3-bis (1,1-dimethylethyl)-, tetrahydrofuran, 2-ethyl-5- methyl-, hexane, 1- propoxy-, tridecane, undecane, 4,7- dimethyl-, hexyl octyl ether, decane, 2,6,6- trimethyl-, and heptane, 2,2,3,3,5,6,6-heptamethyl-. The main VOCs produced by Bt851 were phenol, 2,4-bis (1,1-dimethylethyl)- 14.28% (RT = 22.17), followed by dodecane, 2,7,10-trimethyl- 9.53% (RT = 18.21), benzene, 1,3-bis (1,1-dimethylethyl)- 8.47% (RT = 14.12), and benzene, 1-ethyl-2-methyl- 8.41% (RT = 6.62) (Table 3).

## Discussion

Due to the importance of extracted sugar from sugar beet, the presence of toxins and chemical residues may endanger people’s health. Therefore, replacing safe methods like biocontrol agents instead with chemical control is a requirement. It is well documented that endophytic bacteria have potential biocontrol activity against plant pathogens. Each plant harbors to a certain degree-specific microbial community, and in some plant–microorganism interactions, the evolutionary relationships have been reported (Bulgarelli et al., 2012; Zachow et al., 2014). Due to the adaptation of endophytic bacteria to sugar beet, such bacteria might provide better effective biocontrol activity. With this hypothesis, endophytic bacteria were isolated from healthy sugar beet and sea beet plants. In this study, we reported that the VOCs produced by some of these endophytic bacteria, including *Streptomyces* sp. B86, *Pantoea* sp. Dez632, *Pseudomonas* sp. Bt851, and *Stenotrophomonas* sp. Sh622, significantly reduced the virulence traits of *B. pumilus* Isf19 both *in vitro* and *in situ*. The effect of VOCs produced by these biocontrol strains on *B. pumilus* strain as a pathogen has not been reported before. The VOCs of biocontrol strains have shown antibacterial activity against other plant pathogens, like the VOCs produced by *Streptomyces coelicolor* and *Streptomyces avermitilis*, respectively, with a broad-range antibacterial activity (Tetzlaff et al., 2006; Zhao et al., 2008). A plant-associated bacteria, *Pseudomonas fluorescens* B-4117, emit volatiles that inhibited the growth of *Agrobacterium tumefaciens* and *Agrobacterium vitis* (Dandurishvili et al., 2011). Similarly, the VOCs of *Pseudomonas fluorescens* strain showed bacteriostatic effects on *Ralstonia solanacearum* (Raza et al., 2016a). Although many studies reported on VOCs produced by *Pantoea* and *Stenotrophomonas* strains with antifungal activity (López et al., 2021), only a few studies reported the effects of VOCs on virulence traits of bacterial pathogens (Ghasemi et al., 2021).

Motility and biofilm formation are major adaptive behaviors for *Bacillus* species in plant tissue colonization and invasion (Houry et al., 2010). In addition, chemotaxis has a major role for plant-associated bacteria, including *Bacillus* species, whether they are beneficial or pathogenic (Allard-Massicotte et al., 2016). Results presented in our study revealed that VOCs produced by endophytic bacterial strains significantly inhibited motility and chemotaxis behaviors of *B. pumilus* Isf19. This result is in line with previous reports that show motility behavior of bacterial cells is controlled by chemotaxis (Sourjik and Wingreen, 2011). Surprisingly, no inhibition effects between VOCs-treated and non-treated *B. pumilus* Isf19 cells were observed in biofilm formation. This finding is in agreement with well documented that there is a motility-to-biofilm transition among bacteria where the inhibition of motility promotes biofilm formation (Guttenplan and Kearns, 2013).

*Streptomyces* species, as a member of *Actinobacteria*, are the most taxa found in the rhizosphere of sugar beet plants (Cordovez et al., 2015). Members of the genus *Streptomyces* produce a wide range of VOCs which are important for the interaction between microorganisms and suppression of phytopathogens (Wan et al., 2008). The VOCs produced by strain *Streptomyces* sp. B86 significantly reduced motility and chemotaxis behavior of *B. pumilus* Isf19. In contrast, no significant effects were shown in biofilm formation and attachment. Two main VOCs produced by this strain were 2-hexyl-1-decanol and 2-methyl-undecane. Togashi et al. (2007) reported that 2-hexyl-1-decanol had a bactericidal effect and membrane-damaging activity against *Staphylococcus aureus.*

Strain Sh622 showed high similarity to *Stenotrophomonas* sp. BG28 species. Several strains of this genus were reported as effective biocontrol agents against many bacterial plant pathogens, such as *Ralstonia solanacearum* (Ryan et al., 2009). López et al. (2021) reported endophyte of tomato plants belonging to genera *Stenotrophomonas* with the antifungal activity which was related to the synthesis of VOCs and soluble compounds. VOCs produced by this strain could significantly inhibit sugar beet root attachment, chemotaxis, and different motility behavior of *B. pumilus* Isf19. Electron microscopic analysis revealed that bacterial pathogen cells were damaged in the presence of VOCs of *Stenotrophomonas* sp. Sh622. The main VOCs produced by strain *Stenotrophomonas* sp. Sh622 were dodecane, 2,6,11-trimethyl and dodecane, 2,3,10-trimethyl. These major compounds may be responsible for antibacterial activity against Isf19. Previous studies have reported the antibacterial activity of these compounds (Rahbar et al., 2012).

Strain Dez632 had high similarity with *Pantoea agglomerans* species. *P. agglomerans* has been reported with antagonistic activity against many bacterial and fungal plant pathogens, which is associated with its ability to antibiotic production or other mechanisms (Dutkiewicz et al., 2016). The genome sequencing of plant beneficial *P. agglomerans* strains indicated the presence of genes involved in the biosynthesis of VOCs that may stimulate plant growth (Shariati et al., 2017). However, there is no information on the involvement of VOCs produced by *P. agglomerans* strains with antibacterial activity. VOCs produced by these bacteria significantly reduced chemotaxis and swimming motility behaviors of *B. pumilus* Isf19. The main VOCs produced by this strain were p-xylene and benzene, 1-ethyl-3- methyl-, and S-methyl methanethiosulfonate. These compounds were produced by bacterial species and were shown to efficiently inhibit plant pathogens (Joller et al., 2020).

GC-MS analysis revealed that bacterial strains tested produce VOCs which had various antibacterial activities. Strain *Pseudomonas* sp. Bt851 significantly reduced chemotaxis, root attachment, and motility but not biofilm formation by *B. pumilus* Isf19. The main VOCs produced by Bt851 were phenol, 2,4-bis (1,1-dimethylethyl)- synonym 2,4-di-tert-butylphenol. This compound was reported in at least 16 bacterial species (Zhao et al., 2020). 2,4-di-tert-butylphenol with antimicrobial activity has been reported from *Pseudomonas fluorescens* and *Pseudomonas monteilii* (Dharni et al., 2014; Ren et al., 2019). Mishra et al. (2020) reported that 2,4,-di-tert-butylphenol isolated from endophytic fungus, *Daldinia eschscholtzii*, inhibited the quorum sensing and biofilm formation of *Pseudomonas aeroginosa.*

*Pseudomonas* and *Pantoea* genera are widely studied as biocontrol agents. Several species within these genera were described to have the ability to increase defense reaction against different plant pathogens (Weller, 2007; Magnin-Robert et al., 2012). Based on results obtained in this study, Bt851 and Dez632 strains showed higher decreasing effects on soft rot development by *B. pumilus* Isf19. Therefore, we decided to examine whether this reduction effect is related to the induction of defense response. We analyzed the expression level of *PR1* and *NBS-LRR2* genes, and markers of the salicylic acid and jasmonic acid pathways, respectively, in sugar beet roots after treatment by Bt851, Dez632, and inoculation with *B. pumilus* Isf19. The expression level of *PR1* and *NBS-LRR2* remained unchanged in roots treated only with endophytic bacteria or after challenge with bacterial pathogen at Day 0 compared to the control. These results are consistent with the finding that plant tissues either did not respond or weakly responded to beneficial bacteria to reduce activation of defense responses which might be important to their successful colonization (Zamioudis and Pieterse, 2012). Our results revealed that *Pantoea* sp. Dez632 and *Pseudomonas* sp. Bt851 can upregulate the SA-responsive gene *PR1* in sugar beetroot slices against *B. pumilus* Isf19. We also showed that Bt851 can stimulate JA-dependent defense, as indicated by higher expression of the *NBS-LRR2* gene upon pathogen challenge, which suggests that at least in part the defense response can be mediated by both SA and JA pathways, activating the expression of *PR1* and *NBS-LRR2* genes independently against *B. pumilus* Isf19. Surprisingly, *B. pumilus* Isf19 could increase the expression of both genes when inoculated into roots. This is in agreement with previous studies that show *Bacillus* species can activate defense reactions in plant tissues (Poveda et al., 2020).

## Conclusion

In conclusion, this is the first report of the effect of VOCs produced by endophytic bacteria isolated from sugar beet and sea beet plants on the growth and virulence traits of *B. pumilus* as the sugar beet root rot agent. The VOCs can spread over a long distance, make bacteriostatic environment around the plant tissues, and therefore would play an important role to keep pathogen away from the roots. Our results clearly revealed that these endophytic bacteria not only reduce bacterial pathogen growth rate, but also restrict its movement to invade plant roots and/or induce plant resistance. *B. pumilus* is a soilborne pathogen that mediates postharvest disease; therefore, the application of such endophytic bacteria in soil and/or storage may be a useful method to increase both the quality and quantity of sugar beet roots. Information on the biocontrol mechanisms mediated by VOCs during microbial interactions is important to develop safer methods to control plant disease.

## Data Availability Statement

The datasets presented in this study can be found in online repositories. The names of the repository/repositories and accession number(s) can be found below: https://www.ncbi.nlm.nih.gov/genbank/ (MZ647525, MZ647528, MZ647535, MZ647537, and MZ647534).

## Author Contributions

SS conducted all the experiments, analyzed the data, and wrote the draft manuscript. BH, BB, and SA assisted and supervised the data. All authors read and approved the manuscript.

## Conflict of Interest

The authors declare that the research was conducted in the absence of any commercial or financial relationships that could be construed as a potential conflict of interest.

## Publisher’s Note

All claims expressed in this article are solely those of the authors and do not necessarily represent those of their affiliated organizations, or those of the publisher, the editors and the reviewers. Any product that may be evaluated in this article, or claim that may be made by its manufacturer, is not guaranteed or endorsed by the publisher.
